# Photocatalytic Inactivation of *Salmonella typhimurium* by Floating Carbon-Doped TiO_2_ Photocatalyst

**DOI:** 10.3390/ma14195681

**Published:** 2021-09-29

**Authors:** Sarunas Varnagiris, Marius Urbonavicius, Sandra Sakalauskaite, Emilija Demikyte, Simona Tuckute, Martynas Lelis

**Affiliations:** 1 Center for Hydrogen Energy Technologies, Lithuanian Energy Institute, 3 Breslaujos, 44403 Kaunas, Lithuania; marius.urbonavicius@lei.lt (M.U.); simona.tuckute@lei.lt (S.T.); martynas.lelis@lei.lt (M.L.); 2 Department of Biochemistry, Faculty of Natural Sciences, Vytautas Magnus University, 8 Vileikos, 44404 Kaunas, Lithuania; sandra.sakalauskaite@vdu.lt (S.S.); emilija.demikyte@vdu.lt (E.D.)

**Keywords:** floating photocatalyst, carbon-doped TiO_2_, thin films, magnetron sputtering, *Salmonella typhimurium*

## Abstract

Photocatalysis application is considered as one of the most highly promising techniques for the reduction in wastewater pollution. However, the majority of highly efficient photocatalyst materials are obtained as fine powders, and this causes a lot of photocatalyst handling and reusability issues. The concept of the floating catalyst proposes the immobilization of a photocatalytic (nano)material on relatively large floating substrates and is considered as an encouraging way to overcome some of the most challenging photocatalysis issues. The purpose of this study is to examine floating photocatalyst application for *Salmonella typhimurium* bacteria inactivation in polluted water. More specifically, high-density polyethylene (HDPE) beads were used as a photocatalyst support for the immobilization of carbon-doped TiO_2_ films forming floating photocatalyst structures. Carbon-doped TiO_2_ films in both amorphous and anatase forms were deposited on HDPE beads using the low-temperature magnetron sputtering technique. Bacteria inactivation, together with cycling experiments, revealed promising results by decomposing more than 95% of *Salmonella typhimurium* bacteria in five consecutive treatment cycles. Additionally, a thorough analysis of the deposited carbon-doped TiO_2_ film was performed including morphology, elemental composition and mapping, structure, and depth profiling. The results demonstrate that the proposed method is a suitable technique for the formation of high-quality photocatalytic active films on thermal-sensitive substrates.

## 1. Introduction

In recent decades, environmental pollution and growing wastewater amounts, in particular, have been recognized as essential issues of environmental management and self-sufficient human life. Conventional wastewater treatment technologies (carbon adsorption, flocculation, activated sludge processes, etc.) use physicochemical and biological treatment methods, which, unfortunately, are not capable of decontaminating all types of viruses, bacteria, fungi, or other harmful microorganisms which can be observed in wastewater [1]. Furthermore, these methods require relatively expensive equipment and, in some cases, can cause secondary pollution. Consequently, various alternatives to conventional wastewater treatment technologies are being developed.

Due to their versatile advantages such as high efficiency, eco-friendliness, and ability to decompose various organic molecules, advanced oxidation processes, and photocatalysis in particular, have emerged as some of the most actively researched and developed green wastewater treatment technologies [2]. Still, photocatalysis involves some challenges regarding its application for real-life wastewater cleaning, and they need to be solved. First, most commonly used photocatalysts (e.g., TiO_2_, ZnO) have relatively wide band gaps and require UV light activation or band gap modification. Second, visible (VIS) light and UV light are strongly absorbed by water (only 20% of initial VIS light flux and about 1% of initial UV light flux remain at the depth of 0.5 m [3]), and for this reason, relatively deep traditional wastewater treatment ponds might be an inefficient solution. Third, the form and/or support of photocatalyst material have to be optimized for the performance within a specific treatment system. For example, powder photocatalysts benefit from a larger specific surface area, but it is difficult to extract them from treated wastewater. On the other hand, photocatalytic coatings on traditional sinkable substrates are convenient for handling, but they usually have a low surface area, lack higher efficiency, and suffer from light adsorption by water columns [4,5]. The floating photocatalyst is a relatively new concept which endeavors to immobilize highly efficient photocatalyst materials on lightweight floating substrates. The application of floating photocatalyst particles for the wastewater cleaning process would overcome the two previously mentioned photocatalysis challenges: light intensity weakening in water, and powder/coating handling issues in real-life wastewater cleaning systems.

Thus far, various scientific works have suggested different methods for semiconductor band gap reduction: anionic doping with nonmetals/metal ions/rare-earth ions [6,7,8], coupling with narrow-band gap semiconductors [9], plasma treatment [10], nanotube formation [11]. Naturally, band gap reduction methods have to be compatible with the selected TiO_2_ synthesis techniques, and in some cases, particularly when immobilization on substrates is used, this might be challenging. For instance, in photocatalysis experiments, the most often used material is anatase-phase TiO_2_. In general, crystallization of the anatase phase requires temperatures of 450 °C or higher. This requirement can be fulfilled by using high-temperature TiO_2_ synthesis techniques together with the calcination step. Typical examples of such methods are sol–gel [11], hydrolysis–precipitation [11], and solvothermal [12] techniques. However, these methods are hardly compatible with temperature-sensitive substrates such as polystyrene, polyethylene, and other polymers, whereas magnetron sputtering (MS) is a low-temperature technique that can be used for anatase-phase TiO_2_ synthesis at near room temperatures. Other advantages of the MS process are the possibility to apply different TiO_2_ doping strategies for the reduction in the band gap, and its high versatility, repeatability, and scalability, amongst others [13,14,15,16].

Recently, the use of floating TiO_2_-based photocatalysts has been considered as an innovative wastewater treatment method. Different types of buoyant substrates and fabrication techniques used for immobilizing TiO_2_-based photocatalysts on the surface of these substrates have been presented in the literature (see Table 1). The predominant fabrication methods of floating photocatalysts are based on chemical synthesis. Meanwhile, the estimation of photocatalytic performance has been carried out using various dyes as contaminants, and only a few authors tested it with microorganisms (e.g., Escherichia coli, bioaerosols). The application of a floating photocatalyst for bacterial inactivation should be further investigated.

In our previous works, we separately demonstrated that MS can be successfully applied for the formation of carbon-doped anatase TiO_2_ films with a reduced band gap [31], and that photo-active catalyst films can be deposited on temperature-sensitive polymer substrates [32,33]. In this work, we combined both approaches and synthesized carbon-doped anatase-phase and amorphous structure TiO_2_ photocatalysts on high-density polyethylene (HDPE) beads by MS, testing them as floating photocatalysts for *Salmonella typhimurium* bacteria inactivation. To the extent of our knowledge, this is the first time such a combination of materials and processes has been analyzed. A thorough material analysis, including elemental composition, structure, morphology, chemical bonds, and bacteria membrane permeability, was performed. Additionally, material stability and potential loss of efficiency were evaluated by cycling experiments.

## 2. Methodology

### 2.1. Synthesis

**Figure 1:**
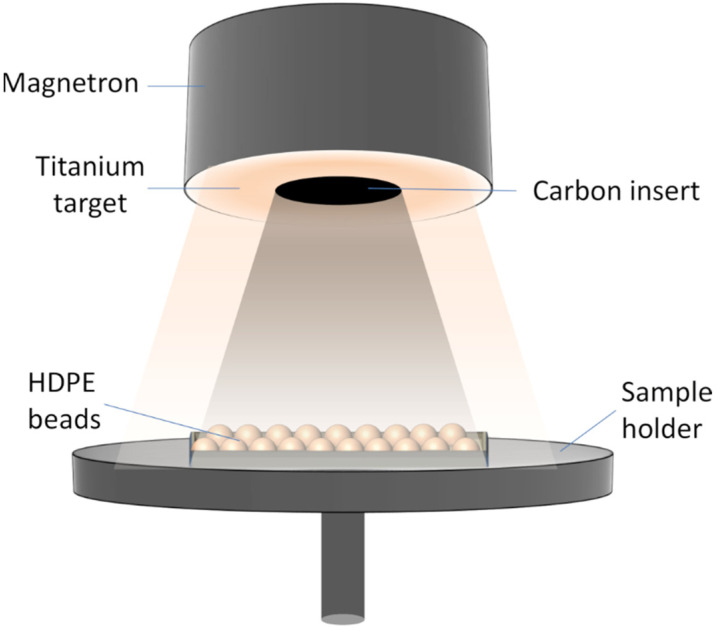
Carbon-doped TiO_2_ formation scheme.

In this study, a physical vapor deposition system (Figure 1) was used to deposit carbon-doped TiO_2_ films on floating HDPE beads (obtained from GoodFellow, Huntingdon, UK). The selection of HDPE as a substrate was justified by its appropriate characteristics: nominal bead size (2–4 mm), density (0.95 g/cm^3^), approximate melting point 130 °C, high durability, chemical inertness, low cost, and potential for recyclability. MS was realized using one magnetron with a high-purity Ti target (95 mm diameter, 99.99% at.). In order to implement TiO_2_ doping by carbon, the central part of the Ti target (50 mm diameter) was partially engraved and replaced by a carbon disc of corresponding size (10 mm thickness) which was cut out from the carbon target (99.9% purity). Prior to the deposition of carbon-doped TiO_2_ films, the base vacuum pressure of 2 mTorr was reached by rotary and diffusion pumps. For the reactive magnetron sputtering process, an Ar (5%)–O_2_ gas mixture was supplied into the vacuum chamber to maintain a constant pressure of 60 mTorr. HDPE beads were placed directly under the magnetron at a distance of 10 cm from the Ti target. The carbon-doped TiO_2_ deposition process was performed using a pulsed DC power supply operating at 260 W (0.7 A) and 300 W (0.8 A). Increasing the power above 300 W caused the melting and destruction of HDPE beads. The total duration of the MS deposition process was 16 h. After the first 8 h of deposition, the HDPE beads were flipped over to deposit the carbon-doped TiO_2_ film on the other side. The approximate thickness of the deposited films was 120 nm at 260 W and 150 nm at 300 W power.

### 2.2. Structural Characterization of the Films

The crystal structure of the samples was characterized by an X-ray diffractometer operating with Cu Kα radiation (XRD, Bruker D8, Hamburg, Germany). Carbon-doped TiO_2_ films were deposited on the flat quartz substrates and HDPE beads under the same conditions simultaneously, and XRD data were collected from flat quartz samples. Crystallite size was estimated by Topas software based on the Scherrer equation with Lorentzian convolution. The surface views of the carbon-doped TiO_2_ were investigated by a scanning electron microscope (SEM, Hitachi S-3400 N, Tokyo, Japan) using a backscattered electron detector. In addition, elemental mapping was conducted using energy-dispersive X-ray spectroscopy (EDS, Bruker Quad 5040, Hamburg, Germany). Surface elemental analysis and elemental distribution profiles in carbon-doped TiO_2_ films were measured by an X-ray photoelectron spectroscope (XPS, PHI 5000 Versaprobe, Chanhassen, MN, USA) using monochromated 1486.6 eV Al radiation, 12.5 W beam power, 50 μm beam size, and 45° measurement angle. XPS depth profile measurement was performed by iterating ion gun sputtering (4 kV Ar^+^ ions, 1 min sputtering time) and XPS spectra acquisition after each sputtering step. Sample charging was compensated by a dual-electron low-energy ion neutralization system and fixing the adventitious carbon C 1 s peak at 284.8 eV.

### 2.3. Bacteria Inactivation

#### 2.3.1. Bacteria Cultivation

The cultivation of Gram-negative *Salmonella enterica* ser. Typhimurium SL1344 bacteria was performed by the procedure described in [34]. The only modification of the procedure was that after dilution to an OD_600_ of 0.15, overnight bacteria culture was grown to an OD_600_ of 0.7 instead of 0.8–1.0.

#### 2.3.2. Bacteria Inactivation Test

Bacteria inactivation experiments were implemented in a temperature-controlled vessel at 37 °C. For each experiment, *Salmonella typhimurium* cells were added to the PBS buffer (pH 7.4). The total volume of the solution was 10 mL. The concentration of bacteria and glucose was 3 × 10^9^ cfu/mL and 0.1%, respectively. The total mass of the floating catalyst was 1 g per experiment. The treatment time for the UV-B lamp (intensity 5 mW/cm^2^, PL-S 9W/01/2p 1CT, Philips, Amsterdam, Netherlands) was 1 h. Two types of control samples (without HDPE beads and light irradiation) were used.

After treatment, the viability of the bacteria was evaluated by the spread plate method. The main parameters of the procedure were as follows: used sample volume—50 µL; sample dilution— 1:2500; incubation medium—LB Agar; incubation time—20–22 h, with an incubation temperature of 37 °C. The remaining part of the treated bacteria suspensions was used for the measurement of membrane permeability by the N-phenyl-1-naphthylamine (NPN) uptake factor assay (more details on the used procedure are provided in [35]).

## 3. Results and Discussion

**Figure 2:**
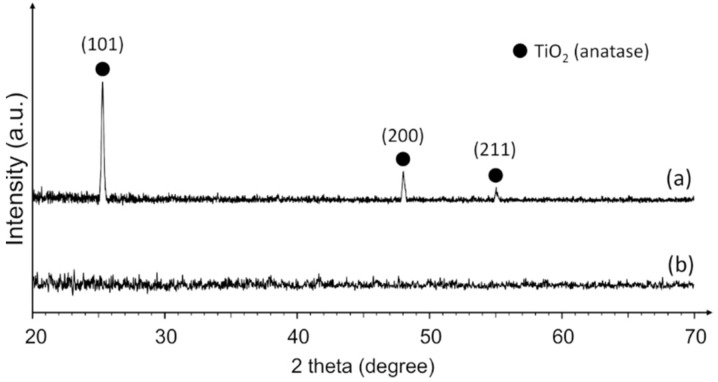
XRD pattern of (**a**) crystalline and (**b**) amorphous carbon-doped TiO_2_ films deposited on the surface of HDPE beads using 300 W and 260 W magnetron power, respectively.

Figure 2 shows XRD patterns of carbon-doped TiO_2_ films deposited on the surface of HDPE beads using 260 W and 300 W magnetron power. MS using a lower power (260 W) resulted in the formation of amorphous carbon-doped TiO_2_. The observation of amorphous TiO_2_ is not extraordinary and, traditionally, is attributed to the too low temperature of the sample during the deposition process which, in turn, depends on the magnetron power. Some of the previous studies reported that temperatures above 300 °C are required for the growth of crystalline anatase instead of amorphous films [36]. However, others suggested that such high temperatures are not necessary and that they might be compensated by other factors. The study of J. Musil et al. revealed a close relationship between the TiO_2_ surface temperature during the deposition process, film deposition rate, and its crystalline phase formation [37]. According to this study, only a 160 °C surface temperature is required to form anatase-phase TiO_2_, but the deposition rate should be relatively low (approximately 5 nm/min). Additionally, it was demonstrated that during the TiO_2_ film deposition process, the actual temperature of the growing surface can be significantly higher (by more than 100 °C) than the temperature of the substrate. These findings comply with our results since a crystalline film was successfully formed using 300 W magnetron power, and the temperature-sensitive HDPE substrate remained stable without any degradation of the structure. Still, excesses of 300 W deposition power significantly increased the HDPE substrate temperature and were responsible for the HDPE melting process.

It was determined that the crystalline phase obtained by 300 W deposition corresponds to the anatase form of TiO_2_ (tetragonal, I41/amd). The average crystallite size was estimated at 38 nm. XRD did not detect any peaks which would be attributed to graphite or any other carbon-containing crystal phase. Additionally, presumably due to the insufficient carbon concentration (see XPS results), the XRD pattern suggests that carbon dopants did not have any significant effects on the anatase structure. This result confirms the formation of a regular TiO_2_ anatase phase which is the most common photocatalytic surface. Similar results were observed by other authors, who used the MS technique for undoped anatase and anatase/rutile TiO_2_ film deposition onto various temperature-sensitive substrates [38,39,40].

**Figure 3:**
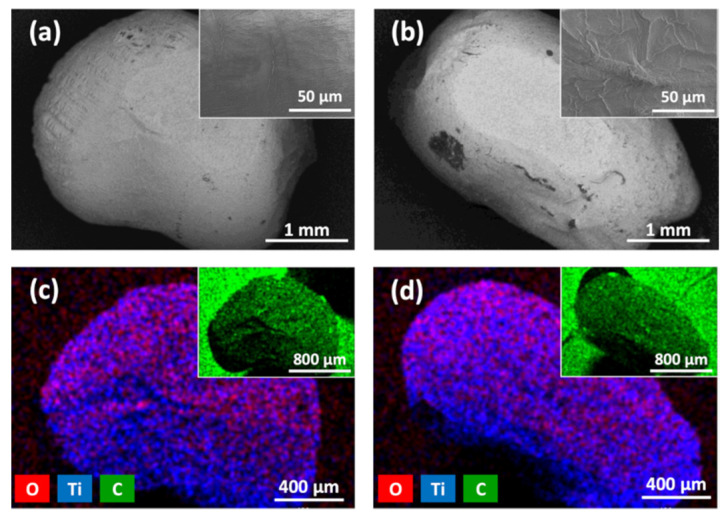
SEM surface images and EDS elemental mapping views of (**a**,**c**) carbon-doped amorphous TiO_2_ and (**b**,**d**) carbon-doped anatase TiO_2_ films deposited on HDPE beads.

Surface morphology and elemental mapping analyses of carbon-doped TiO_2_ films deposited on HDPE beads were performed using SEM and EDS techniques, and the results are shown in Figure 3 (a and c amorphous, and b and d anatase-phase carbon-doped TiO_2_). The SEM surface images (Figure 3a,b) reveal that both the amorphous and anatase-phase carbon-doped TiO_2_ films repeated the HDPE surface texture. However, some cracks and slivers were observed as well (inserts of Figure 3a,b). A slightly higher concentration of cracks and slivers can be observed by analyzing the anatase-phase carbon-doped TiO_2_ films (Figure 3b) compared to the amorphous TiO_2_ films (Figure 3a). This might be related to the higher MS deposition process power and higher HDPE surface temperature. Our previous studies revealed that temperature-sensitive polymers such as EPS or HDPE have a very clear temperature limit. Below these limits, polymers remain remarkably stable, but even a very small increment in the temperature above the limit can cause polymer shrinkage or induce other structural changes [32]. This study reveals that with the used experimental setup, the HDPE temperature limit is reached slightly above 300 W magnetron power. Therefore, we presume that the increase in cracks in the anatase phase of carbon-doped TiO_2_ films indicates the approach of the specific temperature limit for HDPE beads.

EDS elemental analysis and mapping of the films were performed in order to identify all the elements and examine their distribution. EDS analysis observed the existence of three elements: carbon, oxygen, and titanium. Although carbon is a dopant and is present in the films, it is also the main element of the HDPE (–(CH_2_-CH_2_)n–)- and carbon-based sticking pad which was used for the immobilization of HDPE beads during SEM/EDS analysis (inserts of Figure 3c,d). Oxygen and titanium showed a relatively uniform distribution over the surface of the HDPE beads (Figure 3c,d), confirming uniform TiO_2_ deposition in both the amorphous and anatase carbon-doped TiO_2_ films.

**Figure 4:**
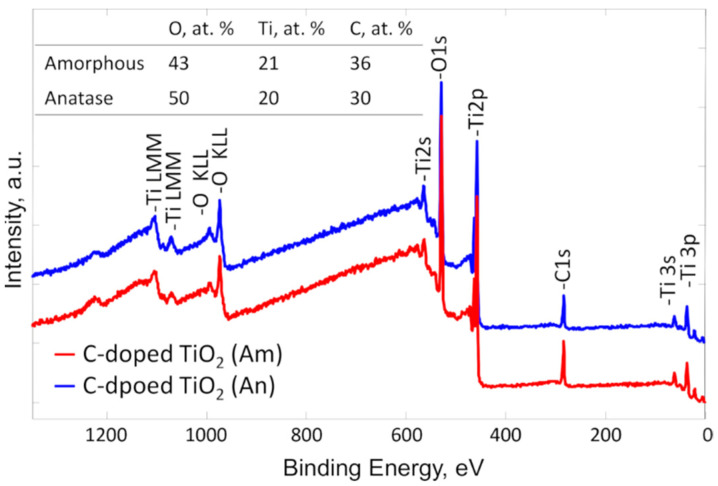
XPS survey spectra and elemental composition of C-doped TiO_2_ amorphous (red line) and C-doped TiO_2_ anatase (blue line).

**Figure 5:**
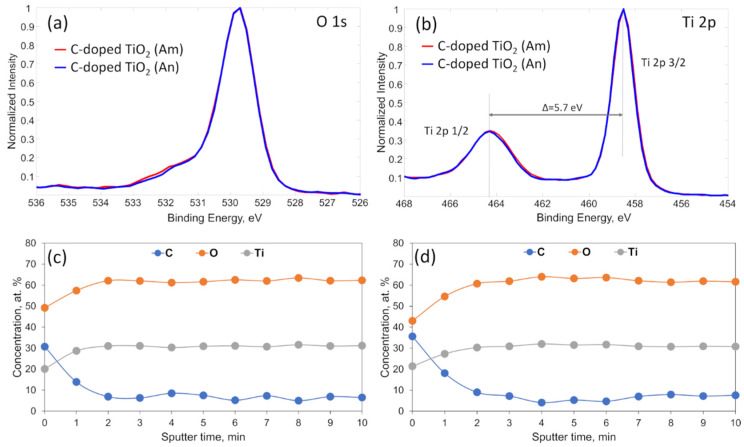
(**a**) O1s and (**b**) Ti2p XPS spectra of amorphous (red) and anatase (blue) carbon-doped TiO_2_ films, and depth profiles of (**c**) anatase and (**d**) amorphous carbon-doped TiO_2_ films.

Similarly, XPS survey analysis also confirmed that the amorphous and anatase carbon-doped TiO_2_ films consisted of the Ti, O, and C elements without any other impurities (Figure 4). Figure 5a,b represent almost identical high-resolution O 1s and Ti 2p spectra of amorphous and anatase carbon-doped TiO_2_. Despite the completely different crystallinities of the samples (Figure 2), Ti 2p XPS spectra consisted of two peaks, Ti 2p 3/2 and Ti 2p 1/2, at 458.6 eV and 464.3 eV, respectively, with 5.7 eV peak separation, which confirms the presence of the TiO_2_ compound in either case [41].

Figure 5c,d include the XPS depth profiles of anatase and amorphous carbon-doped TiO_2_ films, respectively. Films were sputtered for 10 min with a 1 min step, and the quantitative distribution of C, O, and Ti through the films was estimated. At the very top of the anatase film surface, the amount of O, Ti, and C was approximately 50 at. %, 20 at. %, and 30 at. %, respectively, while the amorphous film surface included 43 at. % O, 21 at. % Ti, and 36 at. % C (Figure 4). The enlarged carbon concentration at the top surface can be attributed to the naturally formed thin layer of adventitious carbon (carbon/hydrocarbon layer due to the exposure to the atmosphere). This can be confirmed by analyzing the O 1s spectra in Figure 5a, which involves both amorphous (red) and anatase (blue) spectra. It can be seen that both films have a shoulder at binding energies between 531 and 533 eV. Still, the amorphous film (red) has a slightly higher shift to the left side than anatase. This shows that the amorphous film includes a higher amount of carbon compared to anatase and confirms the result, which was observed at the top layer by performing depth profile measurement. The O 1s components at this region are generally attributed to the various carbon oxide bonds and adsorb moisture [42]. In deeper layers of the films, the O and Ti concentrations increased slightly, and the O/Ti ratio reached a nearly stoichiometric value of 2. The depth profiles confirm that in situ carbon doping resulted in a sufficiently homogeneous carbon distribution, varying between approximately 5 and 7 at. % in both the anatase and amorphous films.

**Figure 6:**
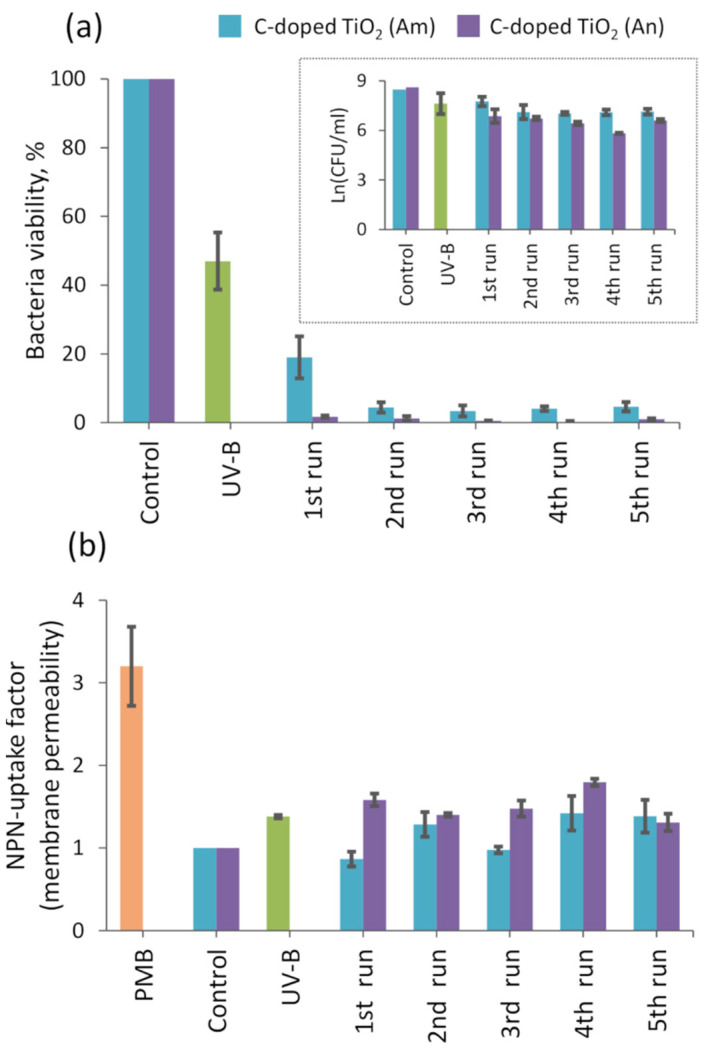
Results of cyclic photocatalytic bacteria treatment by floating carbon-doped TiO_2_ photocatalysts under UV-B irradiation: (**a**) *Salmonella typhimurium* SL1344 bacteria viability; (**b**) NPN uptake factor (membrane permeability).

The photocatalytic activity of the carbon-doped TiO_2_ floating photocatalysts was estimated by measuring the viability of *Salmonella enterica* cells in an aqueous solution under UV-B irradiation (Figure 6a). The influence of UV-B irradiation on bacteria without any photocatalyst showed that viability decreased by approximately 50% after 1 h of exposure. It is important to mention that this value remained relatively stable even after a longer treatment time. Meanwhile, during the first run with UV-B light and floating carbon-doped TiO_2_ photocatalysts, bacteria viability decreased to approximately 19% and 2%, for the amorphous and anatase phases, respectively. Consecutive tests with the same photocatalyst and a new dose of bacteria solution showed an even higher *Salmonella enterica* inactivation rate, displaying reduced viability at approximately 1–3%. These values remained stable in all further runs, exposing our synthesized floating photocatalyst potential for multiple bacteria inactivation applications. O. Akhavan et al. investigated bacteria inactivation and their proliferation after the destruction process using TiO_2_-based photocatalysts [43,44,45]. They showed that the sufficient viability of bacteria is 10% to resume their proliferation. On the other hand, viability of less than 10% stops bacteria proliferation and has practical importance in general.

Additionally, to distinguish the difference between the results using amorphous and anatase carbon-doped TiO_2_ photocatalysts, we calculated the total amount of bacteria in our samples (CFU/mL) and compared the log reduction in the pathogen (insert in Figure 6a). The obtained results of five consecutive runs show increasing bactericidal efficiency of carbon-doped TiO_2_ (An) during the first three runs and its stabilization for the last two runs. Meanwhile, the carbon-doped TiO_2_ (Am) effectivity against *Salmonella enterica* bacteria was stable during the first three runs, and the highest effect was obtained during the fourth run. The fifth run was less effective but not worse than the first three. In comparison with control samples, carbon-doped TiO_2_ (Am) decreased the viability of *S. enterica* cells by 1.5 log and carbon-doped TiO_2_ (An) by 2.5 log. This means that the reduction in the pathogen was approximately 90% and 99% for the amorphous and anatase carbon-doped TiO_2_ photocatalysts, respectively. The bactericidal effect induced by UV light-irradiated undoped anatase TiO_2_ deposited on the HDPE beads was investigated in our previous paper [35]. In the case of undoped TiO_2_, the inactivation efficiency of bacteria averaged 97%, which is higher than the amorphous but lower than the anatase carbon-doped TiO_2_ photocatalyst. Moreover, after the first run, bacterial viability was 6.3% with undoped TiO_2_, while viability decreased to 1.7% with anatase carbon-doped TiO_2_. Generally, it can be stated that the obtained photocatalytic performance is more or less similar or even higher in comparison with that achieved by other authors (Table 1).

Additionally, we determined the permeability of the *S. enterica* bacteria membrane after bacteria treatment by UV-B irradiation and TiO_2_ photocatalysts (Figure 6b). Polymyxin B (PMB) was used as a control value for the dead cells. The NPN uptake factor in that sample was approximately 3, whereas in a control sample, it was equal to 1. UV-B irradiation alone increased the NPN uptake factor value up to 1.3. The calculated average values of membrane permeability using UV-B-irradiated carbon-doped anatase-phase TiO_2_ (An) and amorphous TiO_2_ (Am) photocatalysts were approximately 1.5 and 1.2, respectively. The value of 1.2 for the carbon-doped TiO_2_ (Am) photocatalyst might be related to the generation of a lower amount of reactive oxygen species (ROS) compared to the anatase-phase carbon-doped TiO_2_ photocatalyst, and to the fact that photocatalyst beads might shield bacteria from the intensive light. Both anatase and amorphous films generate external ROS, which cause the production of intracellular ROS. These internal ROS are the cause of bacteria degradation. On this topic, the following article may be useful [33]. DCFH-DA is a cell-permeant reagent fluorogenic dye that measures hydroxyl, peroxyl, and other ROS activity in the cell. We are not able to specify which exact group of ROS is formed in bacteria and the reason for its degradation. Still, both types of photocatalyst demonstrated their potential to be used for the successful inactivation of *S. enterica* bacteria from polluted water even after five cycles.

## 4. Conclusions

This study presents the results of carbon-doped TiO_2_ thin film deposition on HDPE beads by magnetron sputtering and their usage as floating photocatalysts for bacteria inactivation. The carbon doping and TiO_2_ deposition processes were performed simultaneously using a customized magnetron target design. Two types of carbon-doped TiO_2_ films were obtained using a pulsed DC power supply operating at 260 W (0.7 A) and 300 W (0.8 A).

With the lower (260 W) magnetron sputtering power, an amorphous carbon-doped TiO_2_ film was obtained. Crystallization is closely related to the sample surface temperature during the deposition process. Accordingly, an increase in the sputtering power is a natural step to enhance the crystallization of a film. However, the conducted experiments showed that there is only a small range of the appropriate magnetron sputtering power (in our case, approximately 300 W) that is high enough for the crystallization of the carbon-doped TiO_2_ film without melting the HDPE beads which are used as a substrate. Except for several cracks, morphology analysis by SEM did not indicate any significant differences between the amorphous and anatase-phase carbon-doped TiO_2_ films on the HDPE beads.

Although XRD observed a completely different structure of the carbon-doped TiO_2_ samples deposited at different power levels, XPS analysis confirmed the formation of the TiO_2_ compound in both cases. The depth profiles of the samples indicated that, throughout the film, the O/Ti ratio was nearly 2, and the average carbon content varied in the range between 5 and 7 at. %.

Amorphous and anatase-phase carbon-doped TiO_2_ floating photocatalysts were used for the *Salmonella enterica* inactivation tests. The viability of bacteria decreased to approximately 19% and 2% after the first run, while all further runs showed viability of 3% and 1% with amorphous and anatase carbon-doped TiO_2_ photocatalysts, respectively. It can be concluded that anatase carbon-doped TiO_2_ showed slightly better inactivation results as well as higher bacteria membrane permeability than amorphous carbon-doped TiO_2_ after five runs. However, both the amorphous and anatase carbon-doped TiO_2_ floating photocatalysts demonstrated their practical ability to be used for bacteria inactivation in polluted water.

## Figures and Tables

**Table 1:** Review of recent studies of floating TiO_2_-based photocatalyst application for the photocatalytic treatment of various compounds.

Substrate	Fabrication Technique	Photocatalyst	Medium/Light	Photocatalytic Performance	Ref.
Cellulose fabric	Magnetron sputtering	TiO_2_	Escherichia coli/UV-LED	100%/1 h	[17]
Pieces of palm trunk	Sol–gel	Salicylic acid-modified anatase TiO_2_	Congo red dye/Sunlight	98.2%/3.5 h	[18]
Fly ash cenospheres	Sol–gel	Fe–N-co-doped TiO_2_	Rhodamine B/Visible light	89%/4 h	[19]
Fly ash cenospheres	Chemical synthesis and calcination	Polypyrrole-sensitized TiO_2_	Methylene blue/Visible light	55%/9 h	[20]
Expanded graphite C/C composites	Sol–gel	Bismuth/nitrogen-co-doped TiO_2_	Diesel/Visible light	83.8%/5 h	[21]
Perlite	Direct precipitation	TiO_2_	Phenol/UV-A	45%/3 h	[22]
Perlite	Chemical synthesis and calcination	TiO_2_ nanoparticles	Furfural/UV-C	95%/2 h	[23]
Perlite	Chemical synthesis and calcination	TiO_2_	Bioaerosols/UV-C	40%/2 h	[24]
Perlite	Chemical synthesis and calcination	TiO_2_ nanoparticles	Ammonia/UV-C	68%/3 h	[25]
Perlite	Sol–gel	B–N-co-doped TiO_2_	Rhodamine B/Visible light	94%/3 h	[26]
Cellulose paper	Dipping and hydrothermal treatment	TiO_2_/Ag_2_O composite	Aniline/Visible light	97%/6 h	[27]
Small pieces of cork	Sol–gel	TiO_2_–polyaniline composite	Methyl orange/Sunlight	95.2%/3.5 h	[28]
Low-density polyethylene	3D printing	TiO_2_	Methylene blue/UV	14%/2 h	[29]
Polystyrene	Strewing solvent casting	Ag^+^-doped TiO_2_	Methylene blue/UV	86%/5 h	[30]

## Data Availability

Not applicable.
